# A rhesus macaque model of α‐dystroglycanopathy caused by a *POMT1* splice altering variant

**DOI:** 10.1002/ame2.70277

**Published:** 2026-08-24

**Authors:** Anya Nordlund, Brian LaMendola, Nathan P. Crilly, Seth Kittle, Betsy M. Ferguson, Anne D. Lewis, Jeff Wall, Samuel M. Peterson

**Affiliations:** ^1^ Division of Comparative Medicine, Oregon National Primate Research Center Oregon Health & Science University Beaverton Oregon USA; ^2^ Division of Genetics, Oregon National Primate Research Center Oregon Health & Science University Beaverton Oregon USA

**Keywords:** α‐dystroglycanopathies, lissencephaly, *Macaca mulatta*, macaque, POMT1, Walker‐Warburg syndrome

## Abstract

**Background:**

Biallelic mutations in genes associated with α‐dystroglycan glycosylation manifest in a spectrum of conditions referred to as α‐dystroglycanopathies, including Walker–Warburg syndrome, which primarily disrupt brain, eye, and muscle development. While small‐animal models for the disease have been developed and described, the condition has not been documented in non‐human primates.

**Methods:**

Three spontaneous cases of severe lissencephaly, microphthalmia, and congenital muscular contracture in infant rhesus macaques were investigated. Histological analysis was performed on brain, retina, and skeletal muscle tissue. Genomic sequencing and RT‐PCR were used to identify and validate the causative pathogenic variant.

**Results:**

The genetic basis for disease was determined to be the result of a rare single nucleotide variant altering a canonical splice site in the *POMT1* gene. Aberrant splicing was confirmed in affected monkeys and H&E staining demonstrated histological markers consistent with severe α‐dystroglycanopathy.

**Conclusions:**

Recapitulation of this clinical disease phenotype in a non‐human primate establishes the utility of rhesus macaques as a model for dystroglycanopathies, such as Walker–Warburg syndrome.

## Graphical abstract

Rhesus macaques homozygous for a naturally occurring spice altering variant in the *POMT1* gene develop lissencephaly, microphthalmia, and muscular contracture. The presentation models severe presentation of the human Walker‐Warburg syndrome.

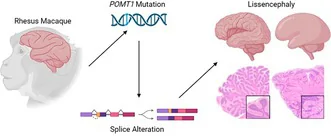

## 1 INTRODUCTION

Walker‐Warburg syndrome (WWS) is a rare, severe genetic disorder characterized by multiple abnormalities primarily affecting the brain, eye, and muscle.^1^ Lesions of WWS appear prenatally, and the condition is often detected in prenatal ultrasounds.^2^ In affected children, WWS is characterized by macrocephaly, microphthalmia, and muscular dystrophy, manifesting as developmental delays and mental retardation with muscular weakness. Commonly identified lesions of the brain include abnormal neuronal migration and development, cobblestone type lissencephaly, cerebellar dysplasia, and hydrocephalus. Observed ocular abnormalities are variable but commonly include microphthalmia, lens defects, and retinal dysplasia with optic nerve atrophy. WWS is a fatal condition, and few patients survive longer than three years.^3^ As an autosomal recessive disorder, it is caused by biallelic mutations in genes associated with glycosylation of α‐dystroglycan (αDG), including *POMT1*, *POMT2*, *FKRP*, *FKTN*, *LARGE*, and *POMGNT1*.^4^

Mutations resulting in altered glycosylation of the αDG protein are referred to as α‐dystroglycanopathies (DGpathies) and are the molecular basis for WWS.^5^ αDG, a cleavage product of the *DAG1* gene, contains a central mucin domain that is heavily enriched for glycosylation target sites and undergoes extensive post‐translational processing.^6^ The glycosylated protein is involved in critical developmental steps, including neuronal migration and patterning, as well as maintenance of skeletal muscle structure. The variable spatiotemporal functions and ligand binding properties of αDG are regulated by alternative processing and the addition of different glycosylation motifs.^7^, ^8^ The multiple post‐translational αDG modification pathways all utilize the same initial step of an O‐mannose group addition catalyzed by the POMT1/POMT2 complex.^9^ Mutations in at least 18 different genes in the αDG glycosylation pathways result in DGpathies, which are classified by phenotype and severity as muscular dystrophy with dystroglycanopathy (MDDG) type A, B, or C and include a range of developmental phenotypes, including mental retardation, brain structural abnormalities, muscular dystrophy, and prenatal or infantile mortality.^10^

Non‐human primate (NHP) models of neurodevelopmental and neurodegenerative disorders are highly translatable to human disease research due to their genetic and physiological similarities with humans.^11^ Rhesus macaques have been a critical biomedical model often used for translational research into infectious disease^12^ and vaccine development.^13^ The genetic characterization of the macaque genome^14^ has expanded the utility of the model organism as a biomedical model for human disease.^15^ Globally, the number of macaque models of human disease generated using genetic modification technologies has increased substantially.^16^ Additionally, collaborative efforts have generated a plethora of genomic resources^17^ aimed at cataloging genetic variation observed in Indian^18^, ^19^ and Chinese origin rhesus macaques.^20^ These tools have been successfully utilized to identify and characterize several natural genetic models of disease.^21^, ^22^

Herein, we describe a spontaneous DGpathy observed in three rhesus macaques manifesting as severe lissencephaly with microphthalmia and muscular contracture. Genomic investigation and pedigree‐based analysis linked these cases to a pathogenic intronic variant in the *POMT1* gene, which is predicted to disrupt canonical splicing. Reverse‐transcription PCR (RT‐PCR) and amplicon sequencing were used to characterize its effect on native splicing and confirm the molecular consequences of the pathogenic variant.

## 2 METHODS

### 2.1 Animals

All animals described in the study were rhesus macaques housed at Oregon National Primate Research Center (ONPRC). Dams were housed in outdoor corrals or shelters and supplied with food ad libitum. Animal care and husbandry was conducted in accordance with federal guidelines and regulations as stated by the National Institutes of Health Guide and the Institutional Animal Care and Use Committee at the ONPRC which is accredited by the Association for Assessment and Accreditation of Laboratory Animal Care, International.

### 2.2 Clinical and pathological examination

Clinical observations and care were provided to dams upon presentation with dystocia. Euthanasia was performed using intravenous sodium pentobarbital on the one fetus that had a heartbeat at delivery by cesarean section. Full necropsies were performed, which included preservation of tissue in 10% neutral buffered formalin as well as biobanking liver samples for subsequent genetic analysis. Fixed tissues were paraffin‐embedded, cut into 3–5 μm thick sections, slide mounted, and stained with hematoxylin and eosin (H&E). All slides were evaluated by two veterinary anatomic pathologists (ACVP diplomates) and scanned at 20× magnification using an Aperio AT2 400 slide scanner (Leica) for image capture. Normal tissues from age‐matched control animals were obtained from the ONPRC Pathology Services tissue archive.

### 2.3 Candidate variant identification

The Macaque Genotype and Phenotype database (www.mgap.ohsu.edu) was used to identify potential pathogenic variants in the affected animals. At the time of analysis, the mGAP repository (release 3.0) included genomic sequencing data from 4115 macaques including over 1600 monkeys from ONPRC. The database included whole genome sequencing (WGS) data for one of the affected individuals (macaque #2, mGAP ID: m07409). Candidate variants were identified using the Variant Search feature which filters loci based on supplied annotations including observed allele frequency, predicted protein impact, pathogenicity predictions, and the genotype of the affected individual. All raw sequence data used to generate variant level genotyping results are available in the NCBI Sequence Read Archive (SRA) as previously detailed.^18^

### 2.4 Variant genotyping and splicing characterization

DNA was extracted from peripheral blood or frozen liver samples using the Maxwell RSC 48 (Promega Corporation). Variant validation was performed by amplifying the target region with PCR (POMT1‐F: 5′‐AGTGTAAAGGGAAGCCACGA‐3′, POMT1‐R: 5′‐ATCTGCGAGTTCTGCCAGAT‐3′). Amplicons were treated with Exo‐CIP (New England Biolabs) and sequenced on a 3730XL DNA Analyzer (LifeTech, Inc) at the ONPRC Primate Genetics Core. Additional genotyping for pedigree screening of the *POMT1* variant was performed using restriction fragment length polymorphism (RFLP) analysis following amplicon digestion with *FauI* (New England Biolabs), and gel electrophoresis.

RNA was isolated from frozen liver samples with the Qiagen DNA/RNA/miRNA Allprep kit on a QIAcube (Qiagen). Sample integrity was confirmed with the Agilent Bioanalyzer 2100. LunaScript RT (New England Biolabs) was used to reverse transcribe RNA using oligo‐dT primers and splice‐site informative amplicons were amplified using cDNA specific primers (POMT1‐cDNA‐F: 5′‐TAAAGGATCCCGGGAGGCAC‐3′ with either POMT1‐cDNA‐R1: 5′‐GATCTCCAGCTGCCGATACC‐3′ or POMT1‐cDNA‐R2: 5′‐CCATCAGGAAGAACGGGAGG‐3′). Splicing alterations were initially qualitatively assessed with Sanger sequencing and visualization of chromatograms. Sequence‐level characterization and relative quantification of alternative splicing events were achieved by multiplexing amplicons with the Illumina 16S Metagenomic Library Preparation protocol and sequencing on a MiSeq (2 × 300 bp) at the ONPRC Primate Genetics Core. Resulting amplicon sequencing data was trimmed using BBMap^23^ and quantified with the AmpSeq pipeline.^24^

## 3 RESULTS

### 3.1 Gross pathology

Three late‐gestation fetal rhesus macaques were identified with similar congenital malformations. The dams of macaques #2 and #3 presented in active dystocia; one fetus was stillborn in the birth canal and the second was euthanized after delivery by cesarean section. Macaque #1 was discovered deceased; however, dystocia was also considered the likely cause of still‐birth due to the pattern of severe, regionally extensive congestion and cranial trauma. All three exhibited severe gross cranial malformation characterized by an enlarged, domed shaped cranium. The cerebrum in all three macaques was severely enlarged with absence of normal gyri and sulci (Figure 1A,B) and possessed a rough cortical texture (cobblestone lissencephaly; Figure 1C,D), as well as severe dilatation of the ventricles (hydrocephalus). In macaque #1 the hemispheres were present only as a thin remnant surrounding massively dilated ventricles, consistent with hydranencephaly. Although not consistent across all three macaques, multiple other congenital malformations were noted grossly including limb contracture (#2 and 3), facial malformation (#1–3), hepatomegaly (#3), cardiomegaly (#3), ocular malformations including microphthalmia (#1–3), and retinal detachment (#3).

**FIGURE 1:**
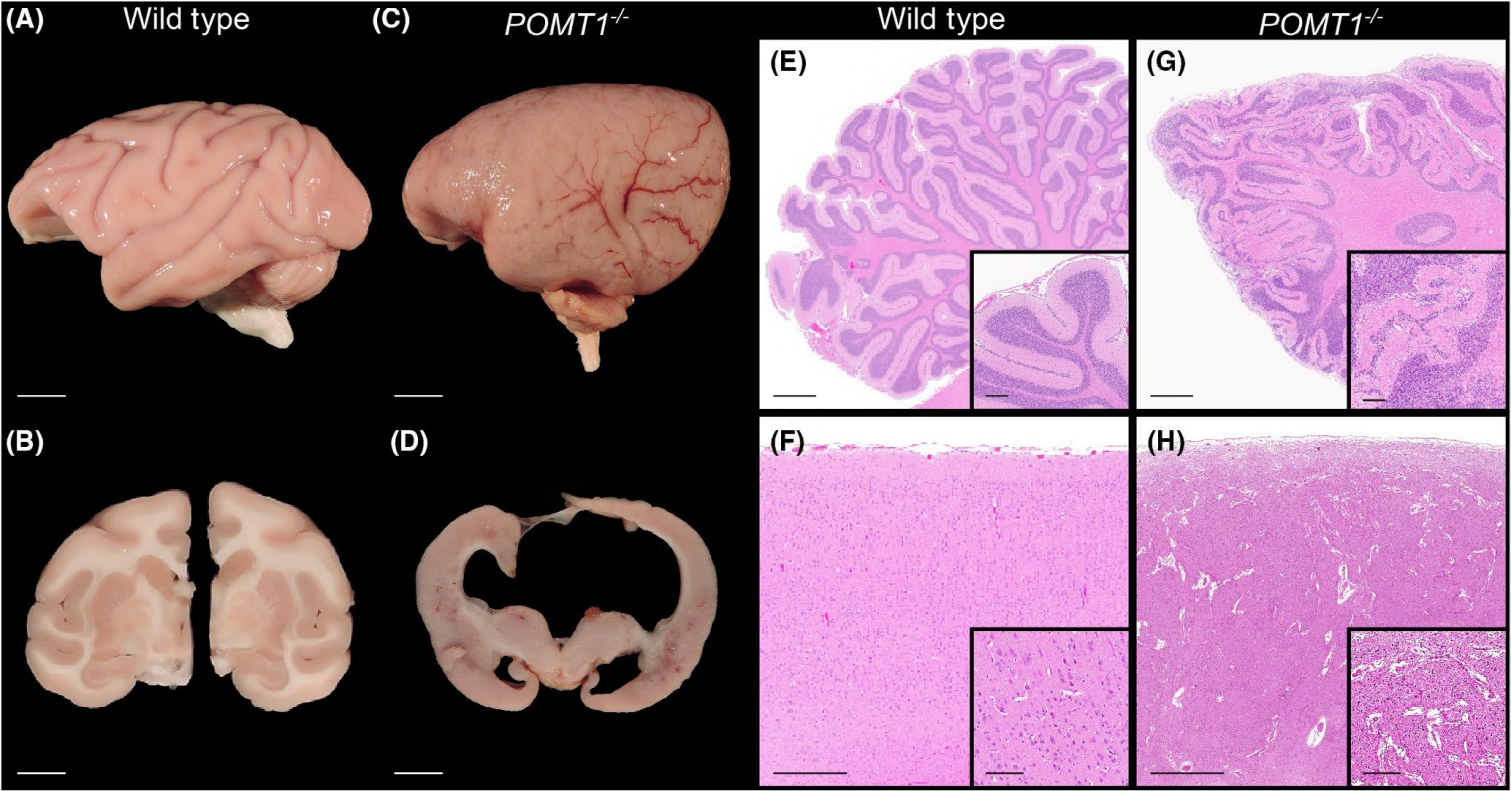
Gross and histologic changes in the brain of a *POMT1* homozygote rhesus macaque compared with wild‐type controls: A‐B) Gross lateral (A) and coronal cross section (B) views of the brain of an age matched control. Gross lateral (C) and coronal cross section (D) views of the brain of *POMT1* ^*−/−*^ macaque exhibiting lissencephaly, hydrocephalus, and cerebellar dysplasia. Normal H&E stained cerebellum (E) and cerebrum (F) from an age matched control. H&E stained cerebellum (G) and cerebrum (H) from *POMT1* ^*−/−*^ macaque. The cerebellum exhibits severe dysplasia with disorganization of the fovea architecture and parenchymal layering. The cerebrum has disorganization of the cortical neuronal layering with increased number and prominence of vascular profiles. Scale bars: 1 cm (A‐D), 1 mm (E‐H main panels), 200 µm (E‐H insets).

### 3.2 Histopathology

Full histopathological evaluations were performed for all macaques. However, macaques #1 and #2 exhibited significant postmortem autolysis which may have hindered discovery and interpretation of more subtle histologic findings. In the brain, all macaques exhibited disorganization of all layers of the cerebral cortex, increased concentrations of glial cells, widening of perivascular spaces, and variable degrees of parenchymal rarefaction consistent with edema (Figure 1E‐H). In macaques #1 and #3, a significant increase in the density of vessels was also observed, consistent with microvascular hyperplasia. Additionally, mild cerebral hemorrhage was noted in macaques #2 and #3 and small amounts of hemosiderin pigment were occasionally associated with the hemorrhage of macaque #2, indicating a degree of chronicity.

Gross ocular malformations were identified in all three macaques; however, severe postmortem autolysis in macaques #1 and 2 prevented histopathologic examination of retinal tissue. Tombstoning of the retinal epithelium in macaque #3 confirmed that the retinal detachment seen grossly was an antemortem change rather than a postmortem artifact (Figure 2A‐D). Both retinas were variably dysplastic with multifocal infoldings and rosette‐like formations, as well as multifocal disorganization and/or absence of the nerve fiber, ganglion cell, and inner plexiform layers. The lens was fragmented, but abnormal orientation of lenticular fibers indicated an underlying degree of lenticular dysplasia.

**FIGURE 2:**
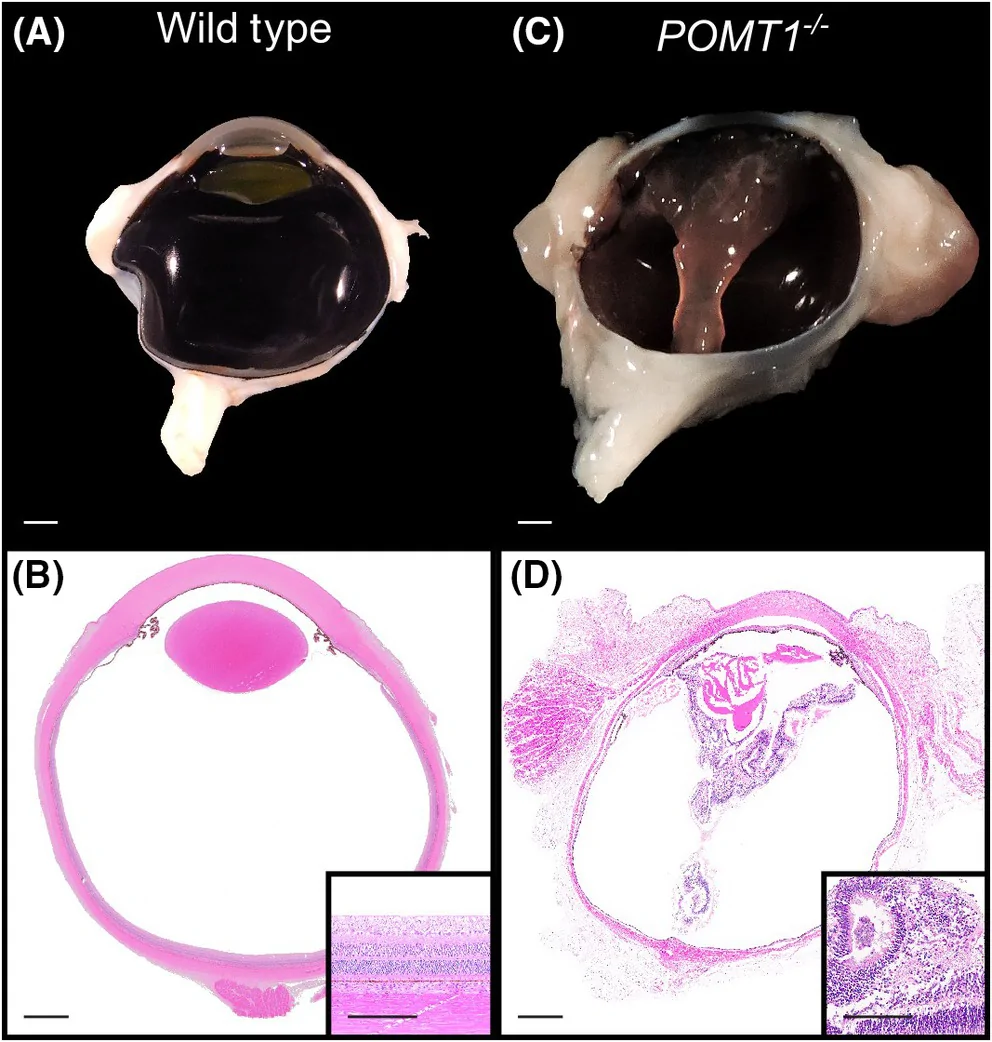
Gross and histologic changes in the eye of a *POMT1* homozygote rhesus macaque compared with wild‐type controls: Gross (A) and H&E stained (B) cross section of a normal age matched control. Inset (B) shows normal layering of the retina. Representative eye (C‐D) from *POMT1* ^*−/−*^ macaque. Gross (C) cross section showing microphthalmia and retinal detachment. H&E stained (D) cross section showing retinal detachment and lenticular fragmentation and malformation. Inset (D) shows rosette‐like invagination and disorganized layering of the retina. Scale bars: 1 mm (A‐D main panels), 200 µm (B,D insets).

Similarly to the eyes, histopathologic examination of musculature was only completed on macaque #3 due to severe postmortem autolysis in macaque #2. The contracted musculature of macaque #3 (Figure 3A) exhibited mild to moderate, multifocal myofiber degeneration and necrosis characterized by myofiber swelling, increased eosinophilic staining, fracture of sarcoplasm, and rarely cell membrane rupture (Figure 3B‐E). Rare myofibers within affected regions exhibited increased basophilic staining and increased numbers of centrally migrating and rowing nuclei, consistent with early regeneration.

**FIGURE 3:**
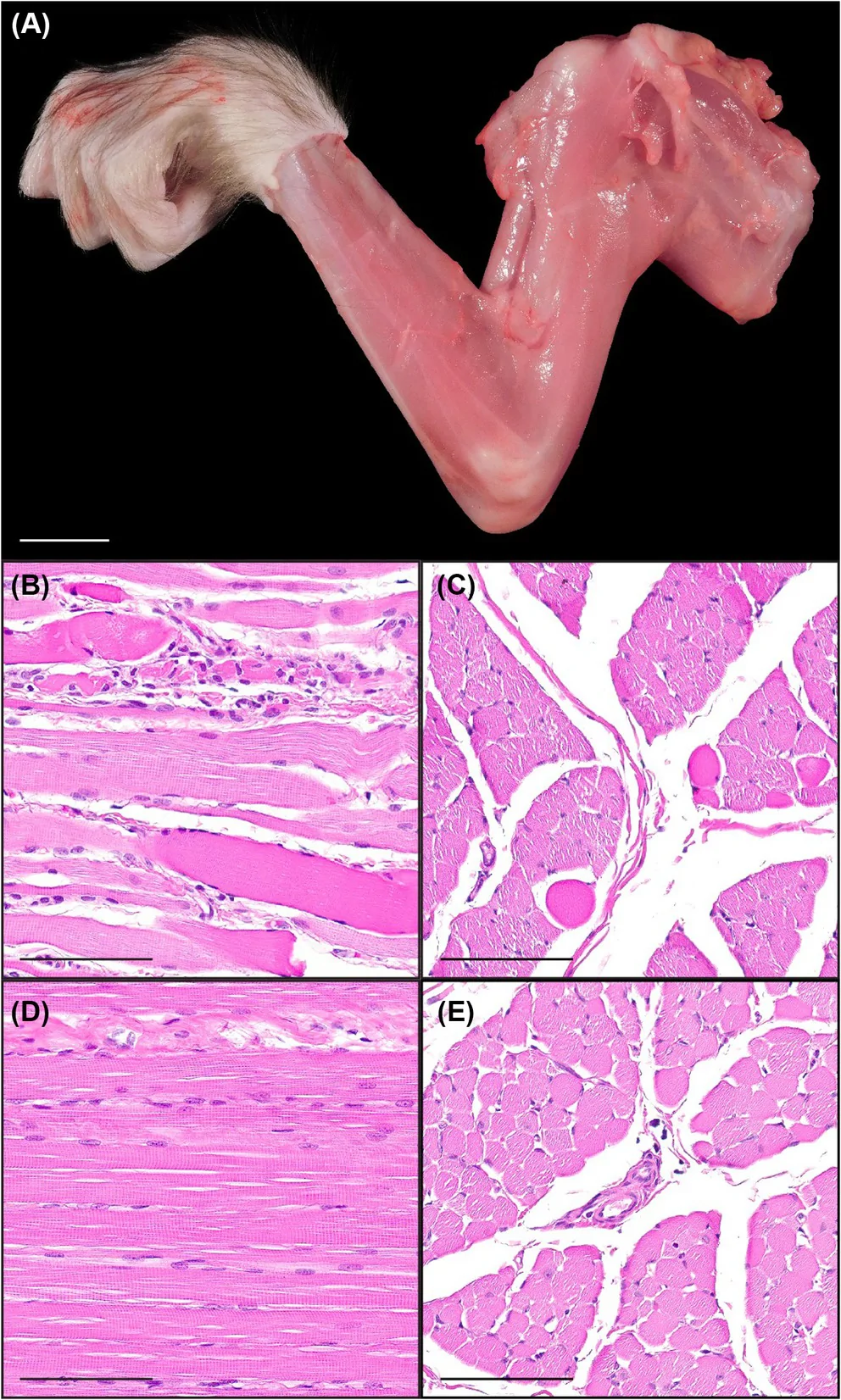
Myodystrophy in *POMT1* homozygote rhesus macaque compared with wild‐type control muscle: Gross (A) image of the left forelimb of macaque #3 with prominent contracture of the brachial and antebrachial musculature. H&E stained longitudinal (B) and transverse (C) muscle from the same animal showing multifocal myocyte degeneration and necrosis compared to stained longitudinal (D) and transverse (E) muscle from an age matched control. Scale bars: 1 cm (A), 100 µm (B‐E).

### 3.3 Identification of pathogenic variant

The shared pedigree and similar presentation of the two lissencephaly cases (macaques #2 and #3) were suggestive of a genetic disorder with an autosomal recessive mode of inheritance (Figure 4A). Since the mGAP database included variant information for one of these individuals (macaque #2; mGAP ID: m07409), an initial query was used to identify 7384 variants for which m07409 was the only observed homozygote, with the majority of these being intergenic single nucleotide variants (SNVs). Based on gene function, known disease associations, and predicted variant impact, a likely causative variant was identified in *POMT1* (c.1273‐2A>G).

**FIGURE 4:**
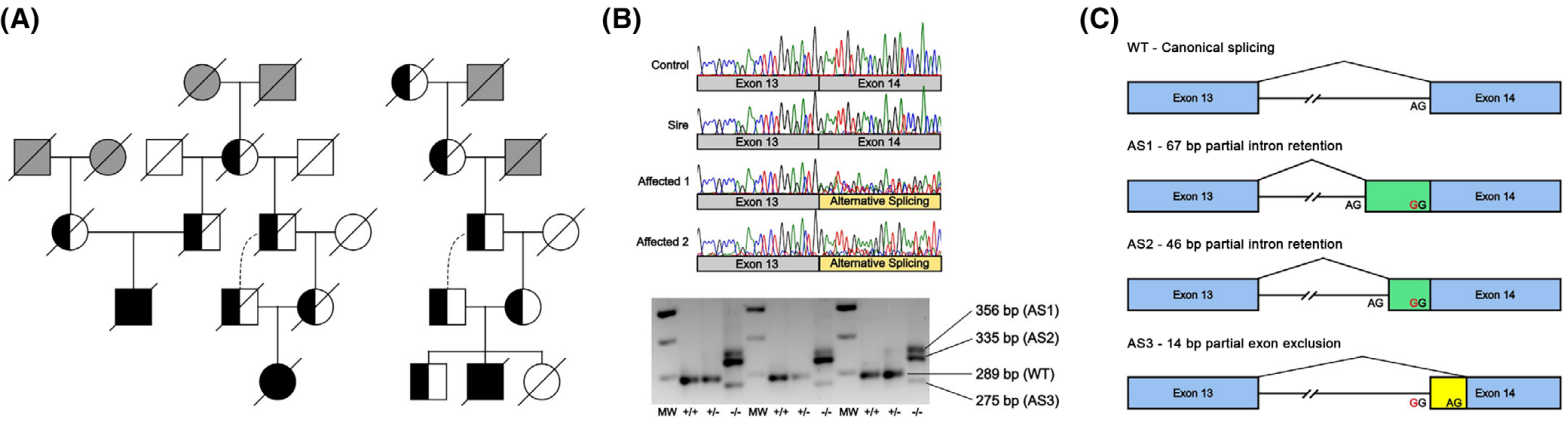
Genetic analysis of *POMT1* mutation. (A) Pedigree of affected individuals reflects an autosomal recessive pattern of inheritance. Genotypes of males (squares) and females (circles) are indicated by shading with a solid black fill symbolizing homozygous, partial fill being heterozygous, solid white being wild‐type, and gray shaded representing unknown genotypes. (B) Chromatograms from *POMT1* cDNA demonstrate that normal splicing observed in wild‐type individuals is perturbed in *POMT1* ^*−/−*^ monkeys after exon 13. RT‐PCR using primers flanking the exon 13–14 junction detected a product of the predicted size (WT) in unrelated controls and heterozygous carriers, while three major aberrantly spliced transcripts (AS1–AS3) were detected in *POMT1* ^*−/−*^ monkeys. (C) Sequence analysis of cDNA amplicons verified canonical splicing resulting from the wild‐type 3′ splice site (AG). In contrast, alternative cryptic splice sites were utilized in *POMT1* ^*−/−*^ monkeys resulting in either partial intron inclusion (AS1 and AS2) or partial exon deletion (AS3).

The candidate causative variant occurs in the splice acceptor site of intron 13 (MMul 10_chr15:8905612) and is extremely rare within the NPRC populations (AF = 0.006). The predicted mechanism of action on *POMT1* transcription is through altering canonical splicing. The rhesus variant is directly homologous to an identified human SNP (rs1296497376), although the mutation has been observed in only one individual in the gnomAD database and only in the heterozygous state.^25^ The corresponding ClinVar^26^ ID (RCV003800866) is listed as being “likely pathogenic” but lacks experimental or clinical support. In silico support of the deleterious potential of the variant is demonstrated by a CADD^27^ score of 35, indicating it is predicted to be in the top 0.05% of all possible human SNPs ranked on deleteriousness. As opposed to coding variants, which can alter the final translated protein product through alteration of the coding sequence, this variant occurs in a canonical splice site (CSS), defined as the two base pairs on either side of an exon. CSS mutations can often lead to protein loss‐of‐function (LoF) and these locations tend to be highly phylogenetically conserved.^28^ The importance of this site is indicated by its extreme species conservation (100 vertebrate PhyloP^29^ score or 8.22) and FATHMM‐MKL^30^ Non‐coding score of 0.972. The splicing‐specific prediction algorithms SpliceAI^31^ and Pangolin^32^ both strongly predict the variant to result in the loss of a native splice site with Acceptor Loss Δ scores of 1.00 and 0.86, respectively.

### 3.4 Variant confirmation

Sanger sequencing was used to validate the candidate variant and confirm the called genotypes of macaque #2 and its parents. Using known carriers identified from mGAP, an expansive pedigree‐based genotyping screen determined that both affected individuals were homozygous for the variant and that the inheritance pattern is consistent with an autosomal recessive disorder. A non‐exhaustive total of 70 carriers were identified, including monkeys housed at other NPRCs. Surprisingly, during this screen, a third homozygote (macaque #1) was identified from an archived sample source. A subsequent review of the individual's Electronic Health Record and necropsy report determined that this monkey was stillborn ~20 years prior and had a presentation consistent with the described phenotype. Given that no unaffected homozygous individuals were identified for the candidate variant, and all other protein altering mutations initially identified from the mGAP search were excluded based on lack of genotype consensus among the three cases, it was determined that the *POMT1* mutation was the causative variant and pathogenic.

### 3.5 Splice utilization

Pedigree analysis and in silico predictions strongly supported the *POMT1* mutation as being the causative variant. However, not all splice site mutations are functional or pathogenic. To confirm the splice‐site altering predictions, we used RT‐PCR and amplicon sequencing to verify and characterize splice altering effects. RNA was extracted from archived liver samples for the three identified cases as well as related carriers and unrelated wild‐type controls. Visual inspection of Sanger sequencing chromatograms from the amplified transcripts clearly demonstrated that transcript sequence alterations were present in the homozygous individuals starting at the exon 13/14 junction (Figure 4B). However, substantial differences in sequence traces of affected individuals and trace degeneracy were suggestive of the presence of multiple alternatively spliced transcripts.

Amplicon‐sequencing was then used to characterize specific alternative splice site usage and quantify the effect of genotype on canonical splicing. Across all analyzed samples, the majority (97.8%) of the identified transcripts mapped to either the canonical splice isoform or one of three novel alternative transcripts. Rare splice events resulting in additional isoforms were at low frequencies across all samples and were excluded from further quantification. As expected, nearly all amplicons (97.0% ± 0.7%, *n* = 6) identified from wild‐type individuals were mapped to the canonical transcript (Figure 4C) while monkeys homozygous for the *POMT1* mutation rarely utilized this splice site (6.1% ± 0.3%, *n* = 3). Interestingly, carriers of the *POMT1* mutation retained mostly normal splicing activity (89.0% ± 4.9%, *n* = 3). Alternatively, the transcripts expressed in homozygous *POMT1* monkeys comprised three main alternative isoforms. Mapping the sequence of these transcripts to the reference genome determined that they resulted from the utilization of alternative splice sites, located either down or upstream of the CSS (Table 1). All three alternative transcripts result from either partial exon exclusion or partial intron inclusion and introduce a premature stop codon, resulting in a truncated protein missing ~300 amino acids. The truncated protein products of the major alternative splicing events are predicted to interrupt the second of three MIR domains and remove two C‐terminal transmembrane domains^33^, ^34^ of the POMT1 protein. These are vital domains to POMT1 structure and likely result in the loss of catalytic activity consistent with a null mutation.

**TABLE 1:** Alternative transcripts resulting from *POMT1* splice site variant.

cDNA Δ	RNA Δ	Protein Δ	Isoform (%)
−14 bp	r.1273_1286del	p.Glu425ArgfsTer34	31
+46 bp	r.1272_1273ins[1273‐46_1273_1]	p.Val727Ter	44
+67 bp	r.1272_1273ins[1273‐67_1273_1]	p.Glu425ValfsTer10	19

*Note*: Transcript descriptions, including predicted RNA and protein changes, are relative to canonical *POMT1* transcript with the total length change being due to either partial intron inclusion or partial exon exclusion. Isoform percentage reflects relative transcript abundance observed in *POMT1* ^*−/−*^ monkeys.

As heterozygous carriers predominantly expressed the canonical transcript, we further sought to phase the splicing events to determine if normal splicing was occurring despite the presence of the mutation in these animals or if transcription was being modified by another means such as nonsense mediated decay (NMD). A longer, alternative amplicon was utilized which simultaneously captures the focal splice site as well as a common missense mutation located in exon 18 (MMul10_chr15:8902364 T>C) which is in linkage disequilibrium with the pathogenic variant. Phasing of transcripts in individuals heterozygous for both genotypes (*n* = 3) determined splice site usage was strongly correlated with the identified pathogenic allele. Almost all (97.9% ± 0.7%) canonically spliced transcripts mapped to the wild‐type allele, while most alternative transcripts arose from the variant allele (95.9% ± 4.3%) indicating that the identified variant strongly influences splicing in heterozygous individuals and that transcript abundance is likely influenced by NMD due to the inclusion of a premature stop codon. Wild‐type individuals who had an exon 18 genotype that allowed phasing demonstrated allelic balance with approximately equal number of transcripts arising from each chromosome (54.1% ± 8.0%; *n* = 4).

## 4 DISCUSSION

WWS and other DGpathies result from genetic variants in the αDG pathway, presenting with a broad phenotypic spectrum including neurological and ocular abnormalities, as well as musculoskeletal deformities. Despite substantial research into the αDG pathway and its role in development,^35^ treatment and therapeutic options are extremely limited. Clinically, genetic screening has been increasingly successful at diagnosing the genetic basis of DGpathies.^36^, ^37^ While there is no treatment or cure available for DGpathies, the use of preimplantation testing in conjunction with clinical and genetic diagnosis has been used to successfully circumvent transmission of *POMT1* mutations following recurrent fetal malformations, highlighting the importance of comprehensive and accurate classification of variant pathogenicity.^38^

This study describes the first known model of DGpathy in a NHP, resulting from a loss‐of‐function variant in the *POMT1* gene. Consistent with the disease spectrum observed in human patients, monkeys homozygous for this mutation were phenotypically similar to descriptions of Walker‐Warburg syndrome^1^, ^39^ which is officially classified with current nomenclature as muscular dystrophy‐dystroglycanopathy (congenital with brain and eye anomalies), type A, 1 (OMIM: 236670). While rare, at least 76 different mutations in *POMT1* have been reported as associated with MGGD with variable severity.^4^, ^40^, ^41^ The variant identified in this study likely results in a complete loss of POMT1 enzymatic activity, which is consistent with severe cases of MGGD in humans not being compatible with life.^42^

Despite the heterogeneity of the disease, available animal models for DGpathies are limited. Knock‐out mouse models for most genes in the αDG pathway have been established.^43^ Loss of αDG glycosylation in rodents is embryonic lethal due to the loss of the murine specific Reichert's membrane which limits their translatability.^44^ To address this complication, alternative approaches have been utilized including tissue specific depletion,^45^ knock‐ins,^46^ or partial knock‐downs.^47^ The pathway has been studied in non‐mammalian models including zebrafish,^48^ fly,^49^ frog,^50^ chicken,^51^ and nematode.^52^

The POMT1 protein is critically vital for αDG glycosylation as it forms an obligate heterodimer with POMT2 to catalyze the initial addition of O‐mannose. As such, perturbations in *POMT1*, *POMT2* and *DAG1* tend to be severe as they affect all downstream processes involving αDG. *Pomt1* knockout mice are embryonic lethal^53^ while tissue specific knockouts develop visual impairments resulting from altered ribbon synapses.^54^ Interestingly, the patterning of retinal layers remained intact in these mice, which is contrary to the results presented in this study. Compared to other models, zebrafish are relatively more resistant to *pomt1* loss which is attributed to the strong effect of maternal mRNA inheritance. However, *pomt1* knockout zebrafish similarly develop disrupted photo receptors, impaired growth, loss of muscle integrity, and eventual lethality,^55^ especially when maternal mRNA inheritance is blocked.

While there have been no previous reports of DGpathy in NHP, CRISPR/Cas9 technology has been used for CMD research to create a Duchenne muscular dystrophy (DMD) model in rhesus monkeys^56^, ^57^ which recapitulates the progressive muscle degeneration and hypertrophic myopathy observed in classic disease pathology. The utility of this NHP model has been demonstrated on two counts: (1) it presents and develops with a similar disease pathology to that observed in humans and (2) it allows the testing of genetic therapies that restore gene expression by exon skipping which improve physiological metrics.^58^ Unfortunately, the role αDG plays in neuronal migration means that the promising results for this approach would likely not translate to this model or other DGpathies where gene correction following development would not restore existing nervous system abnormalities.

The NHP model presented in this study displays phenotypic characteristics consistent with severe, early‐onset human DGpathies, including complex neurological, ocular, and musculoskeletal deformities. While this accurate phenotypic recapitulation highlights the translatability of the NHP disease model, its severe pathology and neonatal lethality have inherent restrictions which limited the scope of characterization and the model's applicability to the study of postnatal disease pathology or therapeutic intervention. The spontaneous presentation and postmortem diagnosis prevented comprehensive tissue collection, limiting histological analysis to a single individual and prohibiting the acquisition of protein‐level data that could have validated perturbed αDG glycosylation. Regardless, the genetic and phenotypic evidence strongly supports the identification of the causal pathogenic variant. Future research applications of this model are influenced by the severity of the phenotype. Since the mutation described is expected to impact the first step in the αDG glycosylation process, homozygous individuals would not be expected to respond to ribitol supplementation, which targets enzymes that act downstream.^59^ Additionally, while pharmaceutical^60^ and small molecule^61^ screens have identified compounds which potentially ameliorate DGpathic symptoms, these would have to be initiated in utero to have therapeutic potential in the described model due to the involvement of CNS development in disease pathology. Instead, this model is informative for prenatal disease pathology and screening and could be utilized to test early‐stage genetic interventions. Further, as the only NHP model of a DGpathy characterized to date, it can potentially identify precise, critical stages in development requiring αDG glycosylation that potential therapies would need to target.

The model described is one of a growing number of new disease models being identified resulting from mutations present in the genetic background of macaque populations. Traditionally, NHP colony management programs promote genetic health by avoiding consanguineous breeding. However, confined living spaces and limited founding population sizes can result in unintended inbreeding. The identification of additional pathogenic alleles is directly applicable to improving captive NHP health, as informed mating decisions can directly consider these genotypes to avoid the production of disease models without research relevance or interest, while advances in cryopreservation allow for the archiving of heterozygous sperm for potential future utility. The identification of the pathogenic variant in this study was highly dependent on the use of population‐level variant data. Pathogenic prediction tools are a valuable resource but are typically focused on the human genome, and the process of lifting‐over variants across species can lead to erroneous predictions. The ability to use allele frequency as a filter, as well as the number of observed homozygous individuals for a genotype in the large NPRC population, is critical to narrowing the list of possible variants to a reasonable number. Given the wealth of genomic data now available, it is likely that the discovery of NHP biomedical models will continue to expand.

## AUTHOR CONTRIBUTIONS

**Jeff Wall:** Resources; funding acquisition; methodology. **Brian LaMendola:** Writing – original draft; investigation; visualization; formal analysis. **Seth Kittle:** Methodology; validation; investigation. **Betsy M. Ferguson:** Conceptualization; resources. **Anne D. Lewis:** Conceptualization; resources; project administration; writing – review and editing. **Nathan P. Crilly:** Conceptualization; writing – review and editing; methodology; formal analysis. **Anya Nordlund:** Conceptualization; writing – original draft; investigation. **Samuel M. Peterson:** Conceptualization; writing – original draft; resources; data curation; visualization.

## FUNDING INFORMATION

This work was supported by National Institutes of Health (grants P51OD011092, R24OD021324 and S10OD034224).

## CONFLICT OF INTEREST STATEMENT

The authors declare they have no conflicts of interest.

## ETHICS STATEMENT

Animals described in this study were housed at ONPRC and all procedures conducted were in accordance with protocols approved by the ONPRC Institutional Animal Care and Use Committee (protocol IP00000367).

## Data Availability

Genetic variant data is available through mGAP (https://www.mgap.ohsu.edu) which includes links to raw sequence data deposited in the NCBI Sequence Read Archive (http://www.ncbi.nlm.nih.gov/sra). Any additional data will be provided upon reasonable request from the authors.
